# Breast Cancer Stem Cells

**DOI:** 10.3390/biomedicines6030077

**Published:** 2018-07-17

**Authors:** Judy S. Crabtree, Lucio Miele

**Affiliations:** Department of Genetics and the Stanley S. Scott Cancer Center, Louisiana State University Health Sciences Center, New Orleans, LA 70112, USA; jcrabt@lsuhsc.edu

**Keywords:** breast cancer, stem cells, Notch, Wnt, Hedgehog, Hippo

## Abstract

Breast cancer stem cells (BCSC) have been implicated in tumor initiation, progression, metastasis, recurrence, and resistance to therapy. The origins of BCSCs remain controversial due to tumor heterogeneity and the presence of such small side populations for study, but nonetheless, cell surface markers and their correlation with BCSC functionality continue to be identified. BCSCs are driven by persistent activation of developmental pathways, such as Notch, Wnt, Hippo, and Hedgehog and new treatment strategies that are aimed at these pathways are in preclinical and clinical development.

## 1. Introduction

Breast cancer is the most common type of non-epidermal cancer and is the second leading cause of cancer-related deaths in women worldwide [1]. In the United States (US), there will be an estimated 260,000 new cases diagnosed and over 40,000 deaths in 2018 as a result of breast cancer [2]. While improved surveillance and early detection have improved breast cancer mortality statistics over the past 30 years, there continues to be unacceptably high incidence, recurrence, and mortality associated with this disease.

For decades, the prevailing theory of cancer initiation and progression has been that cancers arise from serial accumulation of genetic mutations in normal somatic cells. These mutations were thought to provide a selective advantage in terms of enhanced proliferation, inhibition of differentiation, and decreased apoptosis. Each subsequent mutation was thought to result in progressive “de-differentiation”, and as mutations accumulated, the cell would regress to a more primitive phenotype. Differentiated cells have a limited lifespan, and such, it was predicted to be a rare event for cells to accumulate sufficient mutations to transform. Once transformed, cancer cells would proliferate indefinitely and form tumors—with each viable tumor cell being equally capable of forming a new tumor.

The model of sequentially acquired mutations in somatic cells is perhaps overly simplistic and in recent years the concept of a cancer stem cell (CSC) has gained considerable traction [3,4]. The cancer stem cell theory was initially documented in acute myelogenous leukemia [5,6]. These studies demonstrated that only a small subset of cells were able to initiate leukemia in mouse models. Serial transplantation studies revealed that CD34^+^CD38^−^ cells had an increased capacity for self-renewal. Further, it was proven that these cells were a definable subset that was consistently clonogenic [5]. This led to the hypothesis that tumors are organized with a cellular hierarchy that is similar to that of normal tissue and are maintained by a small subset of cells that are responsible for tumor formation and growth [7]. CSCs have several key characteristics that are similar to normal tissue stem cells, including self-renewal (i.e., the ability of a cell to renew itself indefinitely in an undifferentiated state), unlimited proliferative potential, infrequent or slow replication, high DNA repair capacity, and the ability to give rise to daughter cells with differentiation potential through asymmetric cell division [8]. However, unlike highly regulated tissue stem cells, CSCs demonstrate dysregulated self-renewal/differentiation programs, are resistant to systemic chemotherapy, and produce daughter cells that arrest in various stages of differentiation [8]. These observations suggest that since the CSC is the only cell type capable of self-renewal, these may be the cells that are responsible for initiating new tumor formation.

## 2. The Study of Breast Cancer Stem Cells

In breast cancer, small populations of cells have been identified and termed breast cancer stem cells (BCSC). Mammosphere formation assays are a popular and convenient way to assess the behavior of these cells. In these assays, BCSCs are stimulated to proliferate by addition of epidermal growth factor and basic fibroblast growth factor to culture media in low adherence tissue culture dishes [9,10]. The cells proliferating under these low adherence conditions give rise to spheroids, which are commonly defined as “mammospheres” and contain a range of stem-cell associated features, including activation of stem cell related signaling pathways [11]. Further, the formation rate of mammospheres correlates well to the tumorigenicity of the parental tissue when measured in limiting dilution mouse xenograft studies [12,13,14]. Because of this, the number of mammosphere-forming cells in a population is thought to reflect the BCSC numbers, while the size of the mammospheres is thought to reflect their proliferative activity. The low plating density of this strategy allows for the BCSCs to proliferate and form non-adherent spheres with limited aggregation, and is more akin to three-dimensional tumor structures than the classical two-dimensional (2D) adherent tissue culture strategies. Mammosphere formation assays can be used to calculate sphere forming efficiency (%SFE) of mammospheres derived from both primary tissue and breast cancer cell lines [9].

Cell surface markers, such as cluster of differentiation 44 (CD44), cluster of differentiation 24 (CD24), and others are also used to identify BCSCs, and fluorescence-activated cell sorting (FACS) allows for BCSCs to be labeled, sorted, and manipulated in the laboratory setting. Initial reports of BCSC identified a subpopulation of CD44^+^/CD24^−/low^/ESA^+^/Lin^−^ cells that were able to efficiently form tumors in nude mice from very few cells. The resulting tumors contained both stem and non-stem cells, suggesting that both self-renewal and differentiation activities were present in the seeding population [15]. In recent years, additional markers have been identified, including aldehyde dehydrogenase 1 (ALDH1), cluster of differentiation 133 (CD133), and cluster of differentiation 49f (CD49f), and the presence of these markers is often associated with chemotherapy and radiotherapy resistance. Therapies targeting BCSCs with these phenotypes are on the horizon [16] and a number of therapeutic antibodies have been proposed that target these biomarkers for the elimination of BCSC. It has also been reported that triple negative breast cancers have the highest expression of these biomarkers when compared to other breast cancer subtypes [17]. Triple negative breast cancers with the CD44^+^/CD24^lo^ phenotype are associated with poor prognosis [18,19].

***CD44:*** Cluster of differentiation 44 (CD44) is a cell surface transmembrane glycoprotein that binds hyaluronan and is involved in many cellular functions, including cellular adhesion, proliferation, survival, and differentiation. BCSCs demonstrate strong expression of CD44 and the presence of this glycoprotein acts to maintain the multipotency of the BCSC population [20]. Because of its elevated expression, CD44 has been a target of breast cancer stem cell therapies [21,22,23].

***CD24:*** Cluster of differentiation 24 (CD24), also known as heat stable antigen (HAS), is a sialoprotein that enhances cellular adhesion, proliferation, and metastasis. CD24 expression is typically very low or absent in BCSC and in vitro studies demonstrated that the upregulation of CD24 inhibited stemness in breast cancer cells [24]. Further, CD24 has been implicated in chemoresistance in breast cancer cell lines, with CD24^+^ cells arising from radiation-treated CD24^−/low^ cells by transmission of genomic instability [25].

***ALDH1:*** Aldehyde dehydrogenase 1 (ALDH1) is a member of the aldehyde dehydrogenase family of proteins that catalyze oxidation of intracellular aldehydes and may have a role in early differentiation of BCSC through its role in oxidizing retinol to retinoic acid. Elevated expression of ALDH1 identifies BCSCs and correlates with poor breast cancer prognosis in receptor negative breast cancers [26,27]. ALDH1 is measured by an enzymatic assay (ALDEFLUOR) and flow cytometry [26]. Inhibitors of ALDH1 have been examined as potential therapeutics, but efforts are hampered by the redundancy of aldehyde dehydrogenase enzymes and the lack of specificity in the small molecule therapeutics [28]. In breast cancer, ALDH1 isoforms thought to be selectively expressed in BCSC include ALDH1A1 and A3 [29].

***CD133:*** Cluster of differentiation 133 (CD133), or prominin, is a cell surface glycoprotein that localizes to membrane protrusions such as microvilli and on the apical surface of some epithelial cells. CD133^+^ BCSC are primarily associated with triple negative breast cancers and they correlate with poor survival [30].

***CD49f:*** Cluster of differentiation 49f (CD49f) is an α6 integrin that homodimerizes with other integrins (CD24 or CD104) to bind laminin and facilitate epithelial cell adhesion to the extracellular matrix. CD49f also cooperates with signal transduction pathways to facilitate communication between the cell and the ECM. CD49f expression is associated with poor prognosis and reduced survival in breast cancer [31].

**CD90:** Cluster of differentiation 90 (CD90) is a GPI-anchored glycoprotein that interacts with integrins on adjacent cells. CD90 is induced by epithelial-mesenchymal transition, and it has been proposed to be induced by immune cells in the tumor microenvironment in BCSC. Lu et al. have shown that the CD90^+^ population in triple negative breast cancer contains BCSC [32,33].

Combinations of these markers have been used to improve prognostic value in the clinic. Recent clinical studies have demonstrated a correlation between triple negative breast cancer aggressiveness and the CD44^+^/CD24^−/low^ phenotype [34]. However, the correlation with other subtypes of breast cancer are less well defined. Heterogeneity of cancer stem/progenitor cells has been well described for leukemia [35], but much more work is required before the landscape of BCSCs is fully understood. Further, breast cancers are intrinsically heterogeneous themselves, with clinico-pathological subcategories based on immunohistochemical staining for estrogen receptor, progesterone receptor, or Her2. More recently, breast cancers have been categorized at the molecular level using gene expression profiling into the additional categories of luminal A, luminal B, basal-like, Her2-enriched, and claudin-low [36,37]. Triple-negative breast cancers include at least 4 molecular subtypes [38]. Remaining questions are whether these subcategories of breast cancer arise from the same or different stem cell lineages. Dontu, et al. [39,40] suggested that different progenitor cells may give rise to the varying subtypes of breast cancer and initial studies of cell surface markers in breast cancer cell lines suggest that, like leukemia, BCSC may have multiple lineages [41,42]. Interestingly, gene expression studies have demonstrated that different breast cancer subtypes may arise from different mammary progenitor lineages, but perhaps not in the way that one might predict [43,44,45]. For example, basal-like cancers arising in women positive for BRCA1 are likely to arise from luminal progenitor cells [43,46]. Further, there is evidence to suggest that BCSCs display a cellular plasticity whereby these cells can transition from epithelial to mesenchymal (EMT) or mesenchymal to epithelial (MET) based on signals from the tumor microenvironment [47].

## 3. Origins of Breast Cancer Stem Cells

The ultimate origin of BCSCs has sparked controversy in the field for many years. It is still unclear whether BCSCs are derived from multipotent mammary stem cells (MaSC), are a unique progenitor population, result from dedifferentiation of non-stem cells, or arise from some combination thereof. The most widely accepted view is that BCSCs arise from MaSC and progenitor cells. Lineage tracing studies demonstrate the presence of unipotent luminal and basal progenitor cells within the developing mammary gland that sustain and support fully differentiated luminal and basal cell lineages, respectively, for long periods of time [48,49]. It is thought that accumulation of mutations in these progenitor cells may give rise to BCSCs since evidence demonstrates similar phenotypic features of CD44^+^/CD24^−/low^ present in both MaSC and BCSC populations [45,47].

Dedifferentiation from non-stem cells is another theory that is supported by recent evidence. Environmental exposure to chemotherapy and radiotherapy leads to genetic and epigenetic alterations in non-stem cells, resulting in de novo generation of BCSC [50]. Changes in the tumor microenvironment can also contribute to the dedifferentiation of non-stem cells to a BCSC phenotype [51,52] and recent evidence suggests MYC-driven epigenetic reprogramming results in the dedifferentiation of mammary epithelial cells to a BCSC-like phenotype [53]. In summary, it appears that “stemness” in breast cancer is a phenotype that can arise through different mechanisms, either by mutation of tissue stem cells or by acquisition of a stem-like phenotype by transformed cells, induced by EMT, chemotherapy, or even targeted therapy. For instance, Bhola et al. [54] have recently shown that in triple negative breast cancer, resistance to mTOR, and/or PI3K inhibitors arises through the emergence of Notch-dependent BCSC. An additional complication is the fact that breast cancers, particularly triple negative breast cancers, can consist of multiple clones that are derived from quasi-Darwinian evolution from an original transformed clone [55]. It is unclear at the time of this writing whether each clone has a separate stem-like population, or whether clones with BCSC can support the survival of other clones without stem-like cells. Single-cell DNA and RNA sequencing will address this question [56], but the field is still in its infancy.

## 4. Signaling Pathways in BCSC

Many signaling pathways are involved in the regulation of normal mammary stem cells, including Notch, Hedgehog, Wnt, and Hippo. In BCSCs, these pathways are deregulated and they may be the mechanisms of tumor resistance, recurrence, and metastasis. There are numerous therapies in preclinical development or in clinical trials that target these pathways either alone or in combination with standard of care, systemic chemotherapy for a variety of tumors, including breast [57,58,59].

## 5. Notch

Notch signaling is a complex, highly conserved pathway that is involved in embryogenesis, differentiation, cell fate determination, proliferation, and maintenance of stem cells. In mammals, there are four Notch receptors, Notch1-4, and five known ligands Jagged1 & 2, and Delta-like1, 3 & 4. Canonical Notch signaling is activated by interaction between the transmembrane ligand and the receptor on a neighboring cell. The Notch receptor is subjected to cleavage by ADAM10 or ADAM17/TACE, which is followed by a final proteolytic cleavage by γ-secretase to release the Notch intracellular domain (NICD). The NICD translocates into the nucleus and binds to a Notch transcriptional complex that includes mastermind-like (MAML) and CSL [60]. This complex activates transcription of Notch target genes, such as the *HES* and *HEY* families of genes, *CYCLIN D1*, and *C-MYC*. Non-canonical Notch signaling has been demonstrated in several cell types, including immune cells, and recent studies of immune infiltration and cancer cell immune evasion are bringing this understudied area to the fore [61,62].

Notch signaling via Notch4 is critical to normal mammary gland development [63,64,65], and Notch expression is present in normal MaSCs [10]. These initial studies suggested that aberrant Notch signaling may be involved in tumor formation through deregulated mammary gland stem cell self-renewal [10]. Inhibition of Notch signaling using pharmacological and genomic approaches reduces the BCSC population and decreases mammosphere formation in breast cancer cell lines, primary cells, and mouse models of breast cancer [66,67,68]. Clinical studies indicate that Notch1 expression is a poor prognostic marker for breast cancer and correlates with BCSC markers, advanced TNM stage, and shorter disease-free survival [69]. Resistance to therapy is thought to be driven by BCSC and the Jagged1-Notch4 interaction was shown to be a key player in ER^+^ resistant breast cancers through BCSC activity [70]. Further, endocrine therapy in luminal breast cancers promotes the self-renewal of CD133^+^ BCSC by inducing a switch from ER-dependent mechanisms to IL6/Notch3 signaling [71]. Notch also mediates EMT by regulating the production of factors, such as Twist, Snail, Slug, and E-cadherin. Notch signaling is often aberrantly activated by hypoxia, such as in the tumor microenvironment or in the stem cell niche, implying a role for Notch in BCSC self-renewal and EMT [72].

## 6. Hedgehog (Hh)

The Hedgehog family consists of Sonic hedgehog (SHH), Indian hedgehog (IHH), and Desert hedgehog (DHH) and it regulates many embryonic signaling processes to control cellular proliferation, fate determination, and patterning [73]. In normal signaling, Hedgehog binds to its receptor, Patched (PTCH1), and releases the inhibitory effect of Patched on Smoothened (SMO). SMO, a G protein-coupled receptor, can then migrate to the cell membrane to be phosphorylated by casein kinase 1 (CK1) and protein kinase A (PKA). This releases the GLI1/2/3 proteins from kinesin family member 7 (KIF7) and the suppressor of fused (SUFU), GLI1/2/3 translocate into the nucleus and activate Hedgehog target genes, such as *CYCLIN D* and *E*.

There are several pieces of evidence suggesting a compelling role for the Hh pathway in BCSC maintenance. Early evidence demonstrated the overexpression of GLI1 in breast cancer cell lines as well as primary breast carcinoma [74]. Liu, et al. demonstrated activation of this pathway in mammospheres with the CD44^+^, CD24^−/low^, Lin^−^ phenotype, and showed that these effects are mediated by the Polycomb gene BMI1 (B cell-specific Maloney murine leukemia virus integration site1) [75]. Other reports show that the Hh signaling pathway is required for maintenance and self-renewal of CD44^+^, CD24^−/low^, and Lin^−^ cells [76,77]. GLI1 and SMO have been shown to be overexpressed in triple negative breast cancers [78], and positively correlate with aggressive tumor stages [78] and CD44^+^, CD24^−/low^ phenotype [79,80]. Hh has also been reported to correlate with increased risk of metastasis and poor patient outcomes [81,82].

## 7. BMI1

BMI1 is a member of the Polycomb family of proteins that function as chromatin modifiers to regulate cell proliferation, embryogenesis, and hematopoiesis [83,84,85]. BMI1 is highly overexpressed in breast cancers [86] and the downregulation of BMI1 in BCSC suppresses tumor growth and proliferation [87]. BMI1 expression confers resistance to tamoxifen in ER^+^ breast cancer [88], and promotes self-renewal of radio- and temozolomide-resistant breast cancer cells [89]. Expression of BMI1 in breast cancer stem cells is mediated by HSP90α through nuclear translocation of c-Myc and EZH2 [90]. The study of BMI1 and other epigenetic regulators, including ncRNAs, has become a very active area of study [91]. Recent work demonstrates the upregulation of miR-494-3p by hinokitiol suppresses BMI-1 expression and inhibits BCSC self-renewal [92].

## 8. Wnt/β-Catenin

Signaling by the Wnt family of proteins is a complex fundamental process to regulate cellular proliferation, cell fate determination, cellular migration, and stem cell niche integrity. Nineteen known Wnt ligands signal through two classes of receptors—the seven-pass transmembrane Frizzled receptors (FZD; a family with 10 members in mammals) and LRP5/6. Canonical Wnt signaling via LRP5/6 and FZD functions by regulating the amount of β-catenin that is available in the nucleus to control gene expression [93], whereas non-canonical signaling pathways are those that are independent of β-catenin and utilize FZD (but not LRP5/6) in conjunction with other coreceptors, such as ROR1/2 [94,95]. When Wnt signaling is off, β-catenin is phosphorylated by a complex, including GSK3, CK1, APC, and Axin and targeted for proteosomal degradation. When Wnt is present, the phosphorylation machinery, including GSK3, CK1, and Axin is recruited to the activated FZD-LRP5/6 complex, releasing β-catenin to translocate to the nucleus and activate gene transcription of Wnt target genes, including *MYC*, *CYCLIN D1*, *TCF1*, *PPARγ*, *AXIN2*, *CD22*, and *COX2.* In mammals, the family of secreted Frizzled-like proteins (sFRPs) and others, can bind to Wnt ligands as functional antagonists that can block canonical and/or non-canonical Wnt signaling. Other antagonists, such as the Dickkopf family of proteins (DKKs), bind LRP5/6 to antagonize only canonical Wnt signaling [16,93]. Studies have shown the Wnt functional antagonist sFRP1 is frequently lost or down-regulated in breast cancers due to aberrant methylation [96], and this loss of sRFP1 is associated with disease progression and poor prognosis [97].

Wnt signaling is a key component of stem cell control in normal tissues [98], and it has been implicated as a key player in cancer stem cells in mammary tumorigenesis. BCSCs with high levels of Wnt/β-catenin signaling are much more tumorigenic than those without [99] and blockade of Wnt/β-catenin signaling suppresses breast cancer metastasis by inhibiting BCSC phenotypes [100]. In vitro, sFRP4, which is a natural Wnt pathway antagonist, chemo-sensitizes BCSCs, and improves the efficacy of doxorubicin/cisplatin chemotherapy [101]. Wnt signaling has been associated with metastasis in triple negative breast cancer [102,103]. Recently, LGR4, a G protein coupled receptor that enhances Wnt signaling through its ligands R-spondins, has been shown to have a role in breast cancer tumorigenesis and BCSC maintenance in mouse models of mammary tumorigenesis [104].

## 9. Hippo

The Hippo signaling pathway is a key regulator in the control of organ size, regeneration, stem cell self-renewal, and tumorigenesis [105,106]. The Hippo pathway is a complex network that consists of a core kinase cascade module that includes MST1/2 and LATS1/2. When activated, this core module results in an inactivating phosphorylation of TAZ/YAP. This phosphorylation event leads to the sequestration of TAZ/YAP in the cytoplasm by 14-3-3, and ultimate proteosomal degradation. Inactivation of the Hippo pathway allows for the translocation of TAZ/YAP into the nucleus for transcriptional activation of target genes [107]. Hippo pathway components have been implicated in mammary gland development in mice, with mouse knockouts of core kinase protein Lats1 revealing absent or lacking epithelial structures in the mammary gland, among other defects [108]. Knockouts of Yap-1 or Sav-1 (an adaptor of Mst1/2) had normal mammary gland development in the virginal state, but displayed hypoplasia, reductions in mammary gland alveolar development, and differentiation during pregnancy [109].

Expression of TAZ/YAP has been reported in breast cancers, including receptor positive, HER2 positive, and triple negative subtypes [110,111,112,113,114,115]. Further, TAZ is correlated with resistance to chemotherapy and increased metastatic activity [110,116,117,118]. Cordenonsi, et al. was the first to perform studies that definitively linked the Hippo pathway, and TAZ in particular, to self-renewal and tumor initiating capability of BCSCs [119]. Subsequent studies using orthotopic mouse models, transcriptomic profiling, and analysis of the transcriptional activation domains of TAZ further supported the association between TAZ and BCSC metastatic ability [110,120].

## 10. Estrogen Receptor α/β

The role of estrogen receptors α and β in BCSCs is still unclear. BCSC are considered ERα negative, but can still be stimulated by estradiol via paracrine mechanisms [121]. Interestingly, a recent study found that ERβ expression is elevated in BCSC, even in ERα negative and triple negative tumor samples. Further, in vitro studies demonstrated that the reduction of ERβ diminishes mammosphere formation in both breast cancer cell lines and patient derived cells [122]. Transcriptional analysis identified a substantial increase in oxidative metabolism upon the suppression of ERβ, an observation that was further demonstrated in vitro, suggesting that the maintenance of BCSC may depend on an ERβ-mediated shift in glycolysis [122]. In other studies, a truncated variant of the ERα, termed ER-α36, has been identified in some ER^+^ breast cancers and has demonstrated to mediate rapid estrogen signaling in BCSC [123]. Further, a recent study in breast cancer cell lines showed that clones carrying mutations in the hormone binding domain of ERα have increased CD44^+^/CD24^−^ ratio, increased mRNA expression of genes associated with stem cell behavior, elevated mammosphere formation activity, and increased self-renewal [124]. These clones also show elevated expression of Notch signaling pathway components and the study suggests that breast cancer cells may acquire stem-like phenotypes through crosstalk between the ER and Notch signaling pathways [124].

## 11. Other Pathways

There is also limited evidence for the involvement of other cell signaling pathways in BCSC function and resistance to therapy. The JAK2/STAT3 pathway has been shown to mediate resistance to tamoxifen in CD44^+^CD24^−/low^ BCSC [125,126], and that the IL6/JAK1/STAT3/Oct4 pathway may be involved in the conversion of breast non-cancer stem cells to BCSC [127]. The TGFβ pathway is critically involved in the stemness and maintenance of MaSC [128,129], and it is also a potent inducer of EMT [130]. In BCSCs, TGFβ and Wnt signaling work in concert and activate EMT to maintain the stem cell fate [131]. Further, the blockade of autocrine TGFβ inhibits the CD44^+^CD24^−/low^ phenotype in mouse BCSCs [132].

## 12. Conclusions and Future Directions

The role of BCSC in cancer initiation, proliferation, metastasis, recurrence, and resistance to treatment is still being understood. Targeting these cells in addition to the bulk tumor cells may have improved efficacy for the treatment of breast cancer and may decrease the incidence of tumor recurrence and resistance to therapy. Several candidate pathways, including Notch, Wnt, Hedgehog, Hippo, and STAT3 have been the starting point for molecular therapies targeting BCSCs, and individual or combination therapies targeting these pathways hold future promise. It must be pointed out that the molecular pathways that control the BCSC phenotype rarely if ever operate individually. These pathways evolved to operate as a network during normal development, and they are linked by multiple redundancies and feedback mechanisms. These pathways form a complex, time-, and dose-dependent functional network that determines cell fate choices. For example, GSK3β, a downstream essential component of the Wnt pathway, is phosphorylated and inactivated by AKT, which is activated by Notch through multiple mechanisms. Vital effector genes, such as *MYC*, *CYCLIN D1*, etc. are transactivated by multiple developmental pathways through super-enhancers responsive to multiple transcription factors. Hence, inhibition of one pathway may trigger compensatory feedback mechanisms that allow BCSC survival by upregulation of other pathways. Rather than envisioning a single “Achilles’ heel” of all BCSC that can be targeted by blocking a single pathway, the functional networks that are responsible for the BCSC phenotype in breast cancer subtypes, or even in individual clones present in each patient, may have to be targeted through rational combinations, adapted to individual patients based on information from ex-vivo models that are used as an aide to treatment planning, such as tumor-derived mammospheres or organoids.
